# Cutaneous candidal infection caused by *Yarrowia lipolytica* in an immunocompetent patient

**DOI:** 10.1016/j.jdcr.2026.03.031

**Published:** 2026-03-20

**Authors:** Kirley Küçük, Carmen Orte Cano, Nefeli Giannopoulou, Blaine Mathison, Marjan Van Esbroeck

**Affiliations:** a Department of Dermatology, Centre Hospitalier Universitaire HELORA, Mons, Belgium; b Department of Dermatology, Hôpitaux Universitaires de Bruxelles, Brussels, Belgium; c Department of Pathology, University of Utah, Salt Lake City, Utah; d Department of Clinical Sciences, Institute of Tropical Medicine, Antwerp, Belgium

**Keywords:** atypical fungal infection, cutaneous candidiasis, immunocompetent patient, opportunistic yeast infection, Yarrowia lipolytica

## Introduction

*Yarrowia lipolytica* is an environmental yeast that rarely causes human infection and is typically reported in immunocompromised hosts. Cutaneous involvement in immunocompetent individuals is highly unusual and may mimic more common infectious dermatoses, making diagnosis challenging. We report a case occurring in an immunocompetent man to highlight this uncommon presentation and the diagnostic difficulties associated with this organism.

## Case presentation

A 56-year-old immunocompetent Armenian man presented with a chronic, pruritic lesion on the dorsum of his right wrist, persisting for 3 years and unresponsive to potent topical corticosteroids. He denied systemic symptoms, recent trauma, or previous similar episodes. His history was notable for frequent handling of a home aquarium without protective gloves and remote travel to rural regions of Armenia and France over 10 years prior.

**Fig 1:**
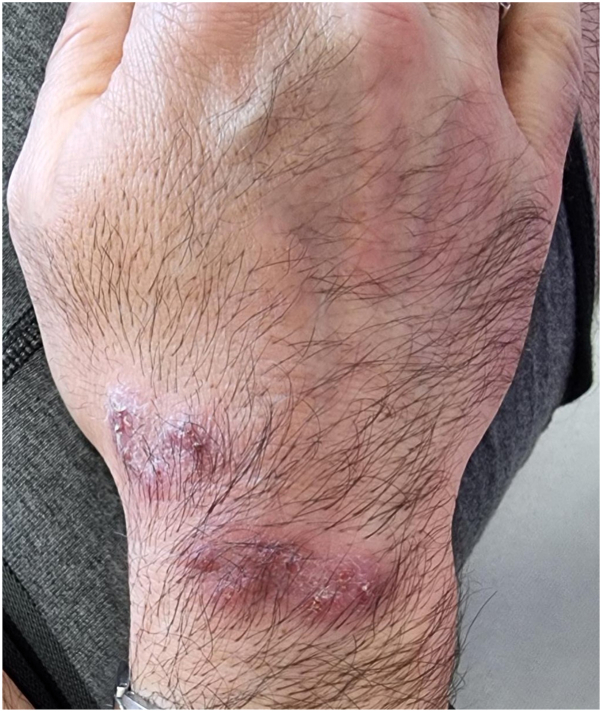
Infiltrated erythematous scaly plaque on the dorsum of the right wrist.

**Fig 2:**
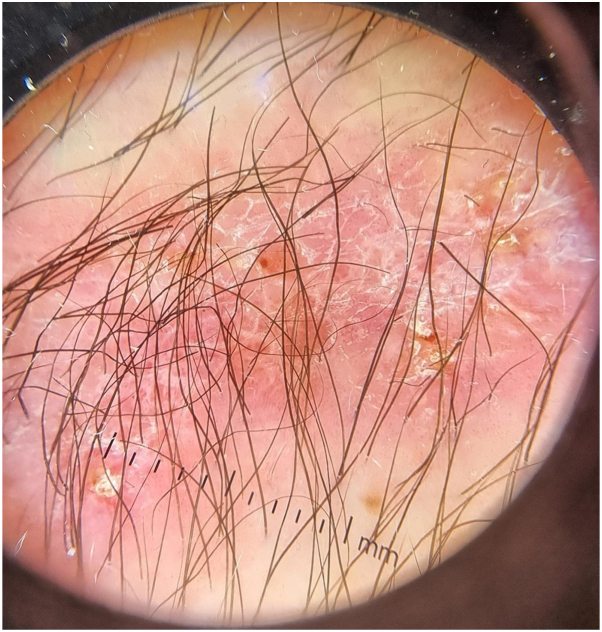
Dermoscopy showed a *pink* background with *white* reticular streaks and *yellow-orange* crusts.

**Fig 3:**
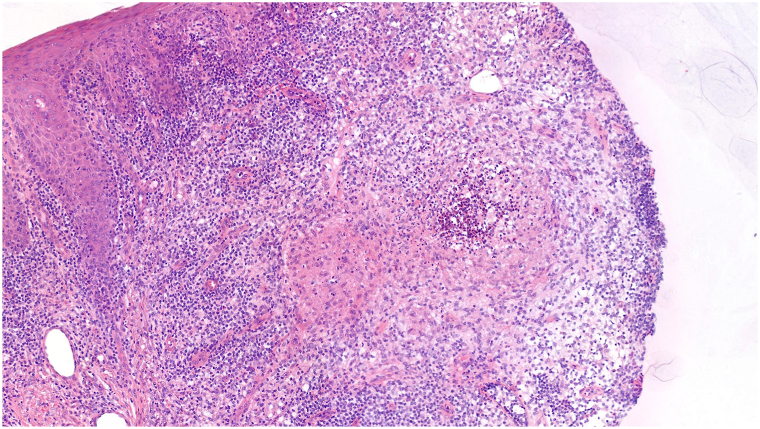
Histological biopsy sample of patient’s lesion on the dorsum of his right wrist showing dermal granulomatous inflammation with focal suppuration.

Physical examination revealed a well-demarcated erythemato-squamous plaque (Fig 1). Dermoscopy demonstrated a pink background with white reticular streaks and focal yellow-orange crusts (Fig 2). A punch biopsy was performed. Histopathologic examination showed dermal granulomatous inflammation with suppurative foci (Fig 3). Ziehl–Neelsen staining and polymerase chain reaction for *Leishmania* spp. were negative. Giemsa staining revealed small budding yeast-like organisms. Fungal cultures on Sabouraud medium yielded creamy colonies that grew more rapidly at 30 °C than at 37 °C. matrix-assisted laser desorption/ionization time-of-flight mass spectrometry confirmed the isolate as *Y. lipolytica*.

The patient was treated with topical miconazole under occlusion. After 1 month, he experienced a near-complete clinical resolution, with significant improvement in pruritus and flattening of the plaque.

## Discussion

*Y. lipolytica*, formerly classified within the Candida genus as Candida lipolytica, is a strictly aerobic environmental yeast. It is widely distributed in industrial and ecological settings, including refrigerated meat, dairy-processing environments, agricultural waste, petroleum-contaminated materials, and soil. Although generally nonpathogenic, it has been implicated in opportunistic infections, particularly in vulnerable or hospitalized populations. Health care-associated cases most commonly involve catheter-related bloodstream infections and suppurative thrombophlebitis. The organism’s ability to produce extracellular slime facilitates adherence to plastic surfaces, particularly intravascular catheters, enabling biofilm formation and subsequent bloodstream invasion in immunocompromised hosts or long-term care patients. These infections tend to occur in clusters and often require catheter removal in addition to systemic antifungal therapy.^1^, ^2^, ^3^

Cutaneous infection due to *Y. lipolytica* is distinctly rare. A limited number of cases have been reported in the literature, occurring in both immunocompromised and immunocompetent individuals with environmental or traumatic exposure.^2^ Only 2 previously published cases describe primary cutaneous involvement in immunocompetent individuals. The first involved a 39-year-old man who developed chronic malar nodules following facial trauma. The causative organism was identified as *Y. lipolytica* after prolonged diagnostic delay.^4^ The second case concerned a 63-year-old woman with chronic obstructive pulmonary disease and hypertension who developed a nonhealing fingertip lesion after a rose-thorn injury. She was successfully treated with a 2-week course of oral fluconazole, with complete recovery.^1^ These limited reports suggest that traumatic inoculation, environmental contact, or repeated exposure to aquatic ecosystems may facilitate cutaneous infection. In our patient, regular manipulation of a home aquarium without gloves likely contributed to inoculation, especially given the chronicity and localization of the lesion.^1^^,^^4^

Diagnosis relies on a combination of histopathology, special stains, and microbiological confirmation. Giemsa stain may reveal yeast-like organisms, but fungal culture is essential for species identification, particularly because *Y. lipolytica* can mimic more common yeasts both clinically and microscopically. Growth at cooler temperatures (around 30 °C) and matrix-assisted laser desorption/ionization time-of-flight mass spectrometry provide reliable diagnostic confirmation.^5^^,^^6^

No standardized treatment recommendations exist due to the scarcity of documented cases. However, azole antifungals have demonstrated effectiveness in the limited reports available. Y. lipolytica generally shows susceptibility to azoles, including fluconazole and miconazole, and in our case, topical miconazole led to rapid and near-complete resolution. Systemic therapy may be considered for refractory, deep, or disseminated infection, but topical treatment appears sufficient for localized superficial involvement in immunocompetent hosts.^1^^,^^5^^,^^6^

This case underscores that *Y. lipolytica*, although traditionally regarded as an environmental and opportunistic organism, can cause primary cutaneous infection even in individuals without immunosuppression. Recognition is important, as the clinical presentation may mimic more frequent etiologies such as chronic candidiasis, cutaneous leishmaniasis, mycobacterial infection, or other granulomatous dermatoses. Definitive diagnosis requires targeted microbiological evaluation, as routine clinical examination and standard stains may not differentiate this organism from more common Candida species.

Dermatologists should consider *Y. lipolytica* in cases of chronic or atypical cutaneous plaques unresponsive to conventional therapies, particularly in the context of environmental exposure. Early recognition facilitates appropriate antifungal management and prevents unnecessary investigations or prolonged morbidity.

## Conflicts of interest

None disclosed.

## References

[1] Boyd A.S., Wheless L., Brady B.G., Ellis D. (2017). Cutaneous *Yarrowia lipolytica* infection in an immunocompetent woman. JAAD Case Rep.

[2] Trabelsi H., Chtara K., Khemakhem N. (2015). Fungemia caused by Yarrowia lipolytica. Mycopathologia.

[3] Kumar S., Kumar A., Roudbary M., Mohammadi R., Černáková L., Rodrigues C.F. (2022). Overview on the infections related to rare Candida species. Pathogens.

[4] Zheng Y.C., Zeng J.S., Li J.W., Wang D.J., Wu Y.Q., Wu Y. (2009). Granuloma caused by Candida lipolytica in China: first case report. J Clin Dermatol.

[5] Pollock C.G., Rohrbach B., Ramsay E.C. (2000). Fungal dermatitis in captive pinnipeds. J Zoo Wildl Med.

[6] Zieniuk B., Fabiszewska A. (2018). Yarrowia lipolytica: a beneficious yeast in biotechnology as a rare opportunistic fungal pathogen: a minireview. World J Microbiol Biotechnol.

